# Cooperative Assembly of Co-Smad4 MH1 with R-Smad1/3 MH1 on DNA: A Molecular Dynamics Simulation Study

**DOI:** 10.1371/journal.pone.0053841

**Published:** 2013-01-10

**Authors:** Guihong Wang, Chaoqun Li, Yan Wang, Guangju Chen

**Affiliations:** College of Chemistry, Beijing Normal University, Beijing, People’s Republic China; Wake Forest University, United States of America

## Abstract

**Background:**

Smads, the homologs of Sma and MAD proteins, play a key role in gene expression regulation in the transforming growth factor-β (TGF-β) signaling pathway. Recent experimental studies have revealed that Smad4/R-Smad heterodimers bound on DNA are energetically more favorable than homodimeric R-Smad/R-Smad complexes bound on DNA, which indicates that Smad4 might act as binding vehicle to cooperatively assemble with activated R-Smads on DNA in the nucleus. However, the details of interaction mechanism for cooperative recruitment of Smad4 protein to R-Smad proteins on DNA, and allosteric communication between the Smad4-DNA and R-Smad-DNA interfaces via DNA mediating are not yet clear so far.

**Methodology:**

In the present work, we have constructed a series of Smadn+DNA+Smadn (n = 1, 3, 4) models and carried out molecular dynamics simulations, free energy calculations and DNA dynamics analysis for them to study the interaction properties of Smadn (n = 1, 3, 4) with DNA molecule.

**Results:**

The results revealed that the binding of Smad4 protein to DNA molecule facilitates energetically the formation of the heteromeric Smad4+DNA+Smad1/3 complex by increasing the affinity of Smad1/3 with DNA molecule. Further investigations through the residue/base motion correlation and DNA dynamics analyses predicted that the binding of Smad4 protein to DNA molecule in the heteromeric Smad4+DNA+Smad1/3 model induces an allosteric communication from the Smad4-DNA interface to Smad1/Smad3-DNA interface via DNA base-pair helical motions, surface conformation changes and new hydrogen bond formations. The present work theoretically explains the mechanism of cooperative recruitment of Smad4 protein to Smad1/3 protein via DNA-mediated indirect readout mode in the nucleus.

## Introduction

Smads, being the homologs of Sma and MAD proteins, as transcription factor proteins to regulate gene expression play a key role in the transforming growth factor-β (TGF-β) signaling pathway that controls a broad range of cellular responses, such as proliferation, recognition, differentiation, migration and apoptosis, during embryogenesis as well as in mature tissues [1]–[4]. Since the TGF-β signaling pathway generally has the antiproliferative activity on many over-proliferated cell types, perturbation of this pathway contributes to several developmental disorders and various human diseases including cancer, fibrosis and autoimmune disease [5]–[9]. There are eight distinct Smad proteins which are subdivided into three classes based on their structures and functions: the receptor-regulated Smads (R-Smads: Smad1, 2, 3, 5 and 8), the single Co-mediator Smad (Co-Smad: Smad4), and the inhibitory Smads (I-Smads: Smad6 and 7) [1], [10]. R-Smads directly interact with activated serine/threonine kinase receptors, and undergo C-terminal phosphorylation. Smad4 acts as the unique Co-Smad in mammalian cells in R-Smads TGF-β signaling pathway by forming heterodimeric R-Smad/Co-Smad complexes that, then, translocate into the nucleus to regulate the expression of target genes [2], [4], [10]–[12]. The crystal structures of Smad3/Smad4 and Smad2/Smad4 complexes have provided useful tools for understanding the important role of Smad4 in the heteromeric Smad protein assembly during the TGF-β signaling pathway [13]. The study on the Smad4 as a tumor suppressor has become an area of considerable interest during the last few decades [14]–[19].

Smad4 and R-Smads, including about 400–500 amino acids in length, share highly conserved structures possessing two globular domains, N-terminal Mad Homology 1 (MH1) domain and C-terminal Mad Homology 2 (MH2) domain, that are connected by a linker of variable length and sequence [3], [12]. The MH1 domain contains sequence-specific DNA binding and nuclear import signaling regions; whereas the MH2 domain is responsible for mediating Smad multimerization and transcriptional activation [20]–[25]. The DNA binding activity of MH1 domain is critical to Smad-mediated function in the TGF-β signaling pathway [20]–[22], [26]–[28]. Since Yigong Shi and co-workers reported the crystal structure of Smad3 MH1+DNA+Smad3 MH1 complex, Prasanna R. Kolatkar and co-workers recently determined the crystal structures of Smad1 MH1+DNA+Smad1 MH1 and Smad4 MH1+DNA+Smad4 MH1 complexes that provide a framework for understanding the final critical step of TGF-β signaling pathway in the nucleus [20]–[22], [29], [30]. These crystal structures reveal in detail that the structures of Smad MH1 monomers are globular with four α-helices (H1–H4) and six short β-strands (β1–β6). Smad1, Smad3 and Smad4 can specifically recognize the same palindromic GTCTAGAC DNA motif termed as Smad binding element (SBE) at the major groove of DNA due to containing the highly conserved β-hairpin motif, and slightly cause overall DNA angle bend in vitro [20]–[22]. In the helix2 of Smad1/Smad3, there is a nuclear localization signal (NLS) which not only is crucial for Smad nuclear import in response to ligand but also is responsible for constitutive nuclear localization of the isolated Smad MH1 domain on DNA [23], [24]. It has been demonstrated that the highly positively charged helix2 of the Smad3 MH1 domain, which contains a lysine-rich nuclear localization signal, plays an important role in the specific contacts between Smad3 and DNA backbone [31]; however, the helix2 of the Smad1 MH1 domain decreases the affinity of Smad1 and DNA in the Smad1 MH1+DNA+Smad1 MH1complex due to its position slightly far away from the DNA interface [21]. Moreover, some studies have revealed that three residues Lys45, Lys46 and Lys48 in the helix2 of Smad4 protein play a central role in nuclear localization signaling for activated R-Smads [25].

Interestingly, recent experimental studies, reported by Prasanna R. Kolatkar and co-workers, revealed that Smad4/R-Smad heterodimers bound on DNA are favorable over homodimeric complexes bound on DNA, which demonstrated that Smad4 protein can act as binding vehicle that cooperatively assembles with activated R-Smads on the TGF-β responsive GTCT elements in the nucleus to regulate the expression of target genes [22]. It has been known from the previous experiments that the DNA shape substantially affects protein–DNA binding through indirect readout mechanism which describes a recognition process that depends on the proper DNA conformation that facilitates its binding to a particular protein [32]; especially, the case that Smad4 protein facilitates the recruitment of R-Smads on DNA is suggestively explained by DNA-mediated indirect readout mechanism [22]. Theoretically, computational and mathematical modeling was used to investigate the dynamics of TGF-β signaling transduction depending on Smad proteins [33]–[36]. The homology modeling of Smad1, 2, 4, 5, 6, 7 and 8 MH1 domains bound on DNA was carried out to analyze functional similarities and differences for these Smad proteins [37]. The molecular dynamics simulations for inhibitory Smads (I-Smads) was employed to investigate the structural flexibility differences between Smad6 and Smad7 proteins [38]. However, computational studies on interaction characteristics of homodimeric R-Smad/R-Smad and heterodimeric Smad4/R-Smad complexes bound on DNA molecule have not yet been detailed at the atomic level so far.

Based on the experimental observations of Smad4 cooperative recruitment function and absence of X-ray structure of ternary heteromeric Smad4 MH1+DNA+R-Smad MH1 complex [22], we prepared, in this study, five independent MD simulations. The first three MD simulations were performed on homomeric Smad1/3/4 MH1+DNA+Smad1/3/4 MH1 complex to investigate their binding properties. The other two simulations were performed on heteromeric Smad4 MH1+DNA+Smad1/3 MH1 complex to address the cooperative assembly characteristics of Smad4 with Smad1/3 on DNA molecule. This study may be helpful for understanding the recruitment mechanism of Smad4 protein by employing DNA-mediated indirect readout mode in the nucleus.

## Models and Methods

### Initial Structures

**Figure 1:**
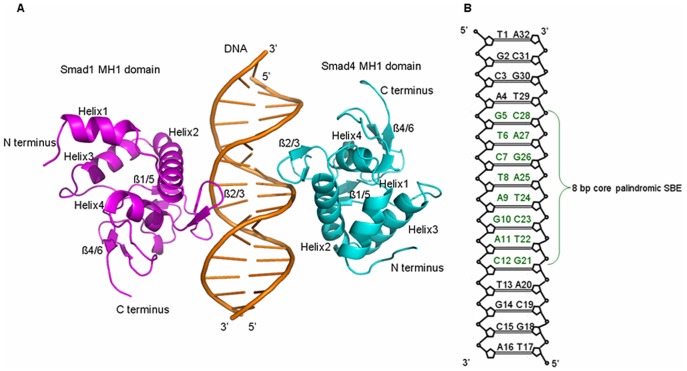
Structure of Smad1 MH1 and Smad4 MH1 bound to DNA and **Sketch of DNA.** (A) The overall structure of Smad1 MH1 and Smad4 MH1 domains bound to SBE palindrome DNA shown in cartoon form with Smad1 MH1 colored in magenta on the left and Smad4 MH1 colored in cyan on the right; (B) Schematic drawing of the 16-bp palindrome DNA sequence with the eight core base pairs colored in dark-green.

Based on the previous experimental studies of Smads, the homomeric Smad4 MH1+DNA+Smad4 MH1 complex as the starting structure for the MD simulation was taken from the X-ray crystal structure (PDB code: 3QSV) (assigned as Smad4+DNA+Smad4 model) [22]. The Smad4+DNA+Smad4 model consists of a 16-bp SBE palindrome DNA fragment, 5′-d (TGCAGTCTAGACTGCA) -3′, and two Smad4 MH1 proteins in which the missing residues, Thr9, Pro139, Gly140 in one Smad4 protein and Thr9-Thr11, Val137-Gly140 in another one, were repaired using the loop search method in the Swiss-PdbViewer program (http://spdbv.vital-it.ch/). Similarly, the homomeric Smad1 MH1+DNA+Smad1 MH1 and Smad3 MH1+DNA+Smad3 MH1 complexes as the starting structures for the MD simulations were taken from the X-ray crystal structures (PDB code: 3KMP and PDB code: 1QZJ, respectively) (assigned as Smad1+DNA+Smad1 and Smad3+DNA+Smad3 models, respectively) [21], [30]. Especially, in order to compare the interaction properties of these similar models, the DNA sequences in the Smad1+DNA+Smad1 and Smad3+DNA+Smad3 models were replaced by the 16-bp SBE palindrome DNA fragment that was used in the Smad4 MH1+DNA+Smad4 MH1 model. Selection of all the coordinates in these two models employed the method of X-ray structures superposing and coordinate importing. All the crystal water molecules were retained as the original coordinates in these models. Other two heteromeric Smad4+DNA+Smad1 and Smad4+DNA+Smad3 models were obtained by using the similar method described above. The structure of Smad4+DNA+Smad1 model in cartoon form was shown in Figure 1A along with the 16-bp SBE palindrome DNA sequence shown in Figure 1B. To compare the differences of conformations between the disturbed DNA molecule and a canonical DNA molecule, a canonical B-DNA molecule simulation was also performed. Given that each strand of DNA has some phosphate groups, 56 Na^+^ and 16 Cl^−^ counterions for the Smad1+DNA+Smad1 model are added to achieve electroneutrality and to satisfy the experimental ionic strength, namely 100 mM for Smad1+DNA+Smad1 complex [29]. Similar counterion processes are applied to other models. The systems were explicitly solvated by using the TIP3P water potential inside a rectangular box large enough to ensure the solvent shell extended to 10 Å in all directions of each system studied.

### Molecular Dynamics Simulation Protocols

All MD simulations were carried out using the AMBER9 package [39] with a classical AMBER parm99 [40], [41] together with the parmbsc0 refinement [42] and gaff [43] force field parameters. The force field parameters and RESP charges of the zinc centers of Smads MH1 were referenced from the references [44], [45]. Details of the MD protocols are given in the Supplementary Materials.

### Free-energy Analyses

The molecular mechanics Poisson–Boltzmann surface area (MM-PBSA) method [46]–[49] in the AMBER9 package was employed to perform the free energy analyses. The binding free energy was computed through calculating the free energy differences of ligand, receptor and their complex as follows:
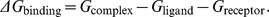



In MM-PBSA, the free energy (*G*) of each state is estimated from molecular mechanical energy *E*_MM_, solvation free energy *G*_SOLV_ and vibrational, rotational, and translational entropies *S*, respectively.
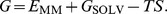


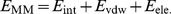


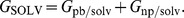
where *T* is the temperature; *E*_int_ is internal energy, i.e. the sum of bond, angle, and dihedral energies; *E*_vdw_ is van der Waals energy; *E*_ele_ is electrostatic energy; *G*_SOLV_ is the sum of electrostatic solvation free energy, *G*_pb/solv_, and the non-polar salvation free energy, *G*_np/solv_. The entropy *S* is estimated by a normal mode analysis of the harmonic vibrational frequencies, calculated using the Nmode module in Amber9 package [50], [51]. Prior to the normal mode calculations, each structure was fully minimized using a distance dependent dielectric of ε = 4r (r is the distance between two atoms) to mimic the solvent dielectric change from the solute to solvent until the root-mean-square of the elements of the gradient vector was less than 5×10^−4^ kcal·mol^−1^·Å^−1^. Then, the entropy was calculated based on standard statistical mechanics expressions [47], [52]. To estimate the reliability of the calculated Δ*G*_binding_ values, we evaluated the standard error (SE) of the calculated binding free energy shifts by referencing the previous works [53]–[55]. Computational details are available in Supplementary Materials.

### Fluctuation and Correlation Analyses

The root-mean-square fluctuations (RMSF) values of residues are a measure of fluctuations and flexibility of backbone Cα of protein over the trajectory broken down by residues in comparison to the average structures [39], [56]. RMSFi of the Cα atom of each residue was calculated as follows:
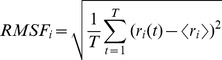
where T is the number of snapshots considered in the time trajectory, r_i_(t), the position of the Cα atom of residue i at time t, and 〈r_i_〉, the time-averaged position of the Cα atom of residue i.

The dynamic feature of a protein and the extent of correlation of the motions of the different regions in a protein were assessed via the calculation of cross-correlation coefficients, C(i,j) given as follows:
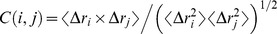
In the equation, Δr_i_ and Δr_j_ are the displacement vectors for atoms i and j, respectively, and the angle brackets denotes the ensemble average [39], [56]. In the present study, the correlation coefficients were averaged over the regions of the protein and the resultant cross correlation coefficients are presented in the form of a two-dimensional graph. These structure analyses in the present work were calculated by using PTRAJ module in the AMBER9 program [39].

### Calculation of Angle between Two Helices

To analyze conformational changes in the relative orientations of any two helices, the program interhlx (written by Kyoko Yap, available at www.nmr.uhnres.utoronto.ca/ikura/interhlx/) was used to calculate the angle between two helices, including the sign in a structure or a family of structures. The program calculates the sign of the angle between two helices by following this convenient role: The two helices are taken to be positioned by helix I being in front of helix II. Helix I (from N to C) is used to define first vertical vector. A second vertical vector is defined with its tail at the C-terminus of helix II. The angle between helices I and II is the rotation required to align the head of the second vector with the N-terminus of helix II. The vector is rotated in the direction that produces an angle of less than 180 degrees with the clockwise or counterclockwise rotation represented by positive or negative sign. This program can also provide other geometry-based parameters such as interhelical distances [57], [58].

### DNA Groove Parameter Analyses

The frequency distributions (fraction of the time spent in each conformation) from the trajectories of simulations for the models and a canonical B-DNA were calculated using the CURVES program [59] to investigate the distortion of DNA. To account for the distortion of the whole DNA backbone, the overall bend, tilt and roll angles of the DNA time-averaged structures for the studied models were calculated from the CURVES outputs using MadBend program [60]. Details of the calculation method are available in Supplementary Materials.

## Results

**Figure 2:**
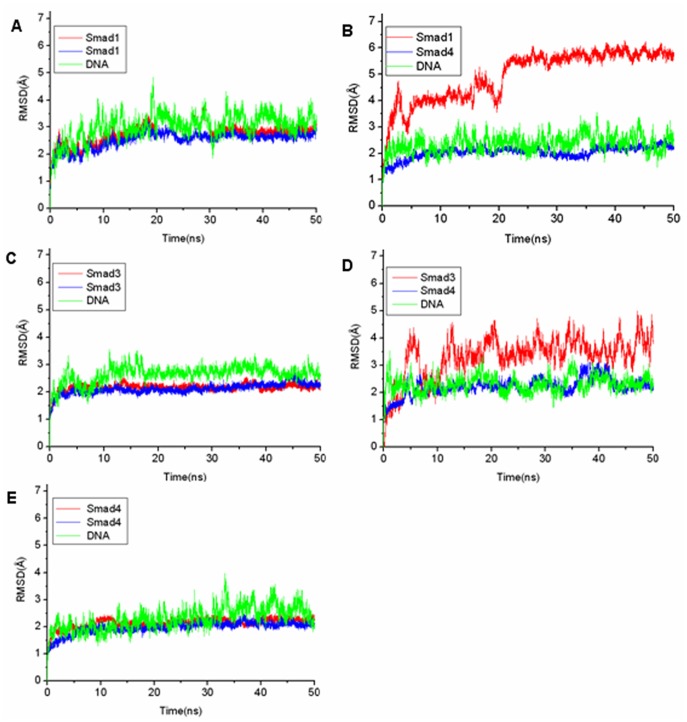
RMSD values of the Smadn+DNA+Smadn models. RMSD values of all backbone atoms with respect to the corresponding starting structures for the simulations of (A) Smad1+DNA+Smad1, (B) Smad4+DNA+Smad1, (C) Smad3+DNA+Smad3, (D) Smad4+DNA+Smad3 and (E) Smad4+DNA+Smad4 models.

The root-mean-square deviation (RMSD) values of all backbone atoms referenced to the corresponding starting structures over all the five trajectories were examined to determine if each system had attained equilibrium. It is often considered that small RMSD values of one simulation indicate a stable state of the system and also suggest that the newly constructed models in this work can satisfactorily reproduce the experimental structures. However, the large RMSD values during a course of simulation suggest large conformation changes of investigated system. Plots of RMSDs of the five system simulations over time are shown in Figure 2. It can be seen from Figure 2 that all the Smadn+DNA+Smadn (n = 1, 3, 4) systems reached equilibrium after 30 ns, and their energies were found to be stable during the remainder of each simulation. Moreover, it can be found from Figures 2B and D that the RMSD values of Smad1/Smad3 MH1 for the heteromeric Smad4+DNA+Smad1/3 system occur certain changes, which predicts the Smad1/Smad3 MH1 allosteric properties. Therefore, the trajectory analysis for the five systems has extracted the equilibrated conformations between 30 ns and 50 ns of simulation time, recording 10000 snapshots at every 2 ps time-interval of each trajectory.

### 1. Interaction and Structure Analyses Revealed Smad4 Cooperatively Recruiting Smad1 on the Binding Sites of DNA

#### 1.1 The calculated data of free energies favor the formation of heteromeric Smad4+DNA+ Smad1 complex

To address Smad4 cooperatively recruiting Smad1, binding free energies for the Smadn+DNA+Smadn (n = 1,4) models were evaluated by using the MM-PBSA methodology, and shown in Table 1 with all energy terms and the total binding free energies with the standard errors. Our results indicate that the Smad1 MH1 domain binding to DNA with the binding free energy of −29.51 kcal·mol^−1^ in the Smad4+DNA+Smad1 model is energetically favorable over Smad4 MH1 domain binding to DNA with that of −17.34 kcal·mol^−1^ in the Smad4+DNA+Smad4 model. Simultaneously, the Smad1 MH1 domain binding to DNA in the Smad4+DNA+Smad1 model also energetically predominates over that with the binding free energy of −18.42 kcal·mol^−1^ in the Smad1+DNA+Smad1 model, which is consistent with the experimental result of −9.60 kcal·mol^−1^ analyzed by the dissociation constant K_d_ for the Smad1 MH1+DNA complex [21]. Consequently, it can be proposed that Smad1 protein may possibly substitute Smad4 in the Smad4+DNA+Smad4 complex, which is consistent with the experimental result that the heterodimer formation on the palindromic SBE effectively competes with the pre-formed Smad4 homodimer [22]. These results support energetically the mechanism of recruitment of Smad4 protein to Smad1 protein reported by the experiments [22].

**Table 1:** MM-PBSA free energy (kcal·mol^−1^) components for the Smadn+DNA+Smadn models.

	Smad4+DNA+Smad4	Smad1+DNA+Smad1	Smad4+DNA+Smad1	Smad3+DNA+Smad3	Smad4+DNA+Smad3
Receptor	Smad4+DNA	Smad1+DNA	Smad4+DNA	Smad3+DNA	Smad4+DNA
Ligand	Smad4	Smad1	Smad1	Smad3	Smad3
ΔE_ele_	−3453.95	−3728.48	−3855.88	−4582.86	−4724.77
ΔE_vdw_	−58.86	−58.27	−53.80	−87.87	−83.84
Δ*E*_int_	0.00	0.00	0.00	0.00	0.00
ΔG_np/solv_	−10.18	−10.49	−10.33	−16.21	−15.61
ΔG_pb/solv_	3445.83	3710.30	3843.88	4577.85	4710.65
ΔG_np_	−69.04	−68.76	−64.13	−104.08	−99.45
ΔG_pb_	−8.12	−18.18	−12.00	−8.01	−14.12
ΔTS	−59.82	−68.52	−46.62	−85.41	−78.01
ΔH_binding_	−77.16	−86.94	−76.13	−109.09	−113.57
ΔG_binding_	−17.34(0.18)^a^	−18.42(0.17)	−29.51(0.18)	−23.68(0.20)	−35.56(0.22)

a The values in parentheses are the calculated standard errors.

Δ*G*_np_ = Δ*E*_vdw_+Δ*G*_np/solv._

Δ*G*_pb_ = Δ*E*_ele_+Δ*G*_pb/solv._

ΔG_binding_ = ΔG_np_ +ΔG_pb_ –ΔTS.

= ΔH_binding_ –ΔTS.

#### 1.2 Hydrogen bond analysis in the Smad4/1+DNA+Smad1model

**Figure 3:**
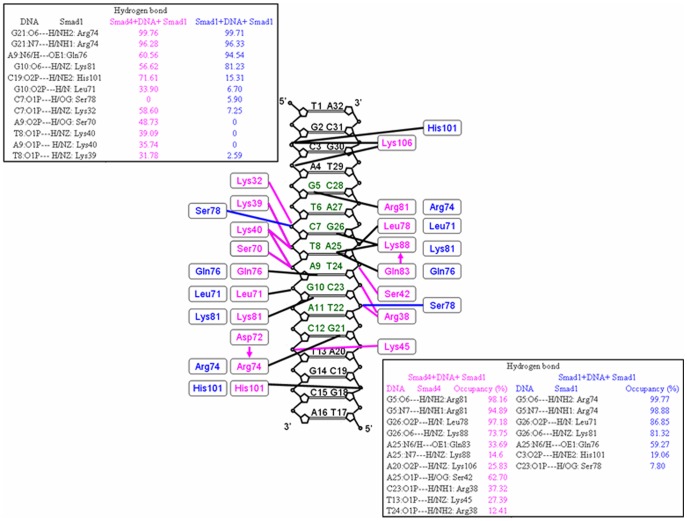
The hydrogen bonds at Smad1-DNA and Smad4-DNA interfaces. The occupancies of hydrogen bonds at Smad1-DNA interfaces in Smad4+DNA+Smad1 (magenta) and Smad1+DNA+Smad1 (blue) models on the left, respectively; and the occupancies of hydrogen bonds at Smad4-DNA interface in the Smad4+DNA+Smad1 model (magenta) and at Smad1-DNA interface in the Smad1+DNA+Smad1 model (blue) on the right, respectively.

It has been found that the Smad4 protein heteromerously binding to DNA increases the affinity of Smad1-DNA interface. To examine the differences of interactions between the Smad4/1 protein and DNA molecule, the occupancies of occurrences of all possible hydrogen bonds at the Smad4/1-DNA interface in the Smad4/1+DNA+Smad1 model were analyzed by calculating the percentages of times during the simulations, and shown in Figure 3, in which the calculated hydrogen bonds for the Smad1+DNA+Smad1 model mostly reproduce the experimental results [21]. Interestingly, there are some additional hydrogen bonds between the residues of helix 2 region of Smad1 protein and DNA phosphate backbone caused by the Smad4 binding for the Smad4+DNA+Smad1 model, compared with the Smad1+DNA+Smad1 model. For example, the formations of additional hydrogen bonds between the N-H groups of residues Lys32, Lys39, Lys40 of helix 2 region of Smad1 protein and O atoms of DNA phosphate backbone at the bases C7, T8, A9 in the Smad4+DNA+Smad1 model are maintained with the occupancies of 58.60%, 31.78% and 35.74% of simulation times, respectively, compared to the Smad1+DNA+Smad1 model. Interestingly, such two newly-formed NH(Lys39)-O(T8) and NH(Lys40)-O(A9) hydrogen bonds induced by Smad4 binding located at the nuclear localization signal region, a lysine-rich motif with its sequence Lys39-Lys40-Leu41-Lys42-Lys43-Lys44-Lys45 in the Smad1 protein, reported by the sequence alignment method using CLUSTALX software [23]. On the other hand, the different hydrogen bonds of Smad4-DNA interface in the Smad4+DNA+Smad1 model from those of Smad1-DNA interface in the Smad1+DNA+Smad1 model were also found, such as hydrogen bonds between the residues Arg38, Ser42 and Lys45 of helix2 region of Smad4 protein and C23, A25 and T13 bases of DNA phosphate backbone maintained with the occupancies of 37.32%, 62.70% and 27.39% of simulation times, respectively. Such different hydrogen bonds are consistent with experimental observations of Smad4-specific contact sites with DNA backbone [22] and are a driving force to modulate Smad1 conformational state favoring DNA binding. Summarily, these results support the binding free energy analysis and the recruitment mechanism of Smad4 to Smad1.

#### 1.3 The structure change at Smad1-DNA interface induced by Smad4 binding

**Figure 4:**
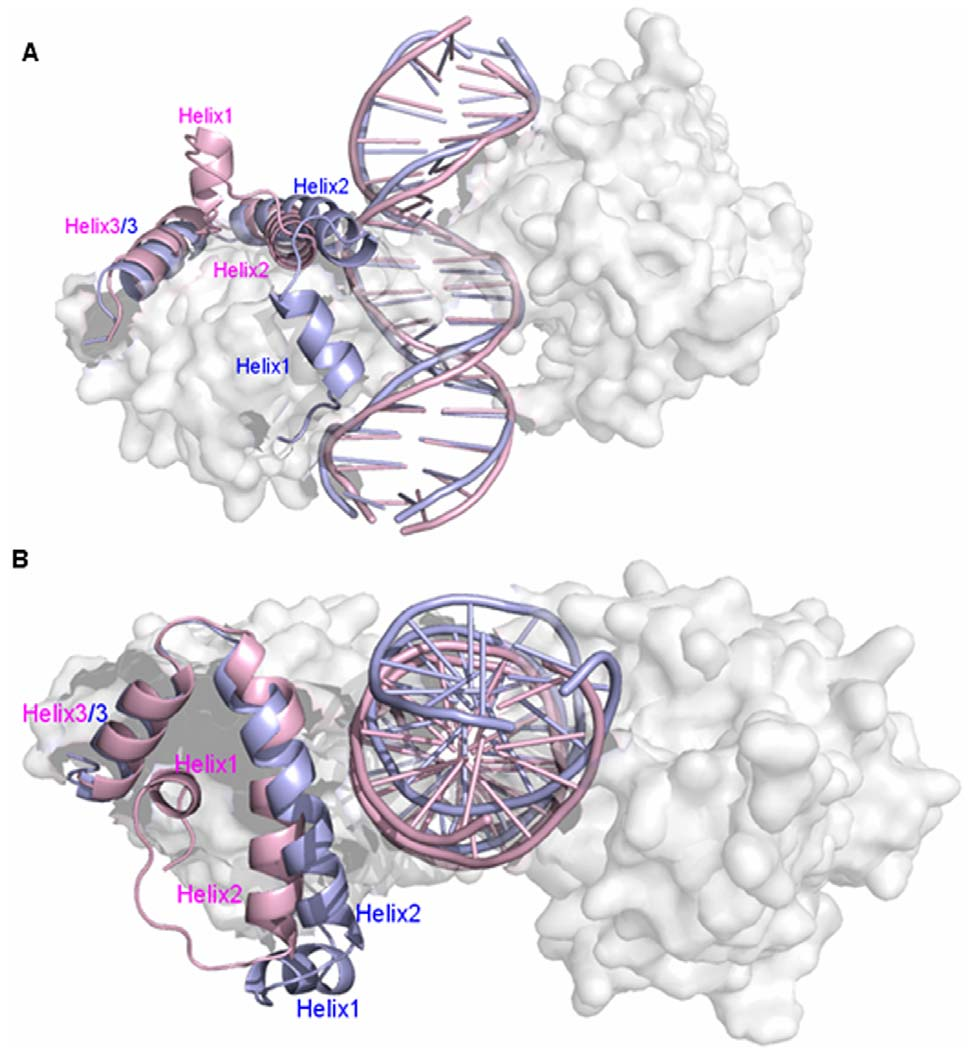
The superpositions of average structures for Smad4+DNA+Smad1 and Smad1+DNA+Smad1 models. The visual superpositions for average structures of Smad4+DNA+Smad1 (light blue) and Smad1+DNA+Smad1 (light pink) models as viewed orthogonal (A) and parallel (B) to DNA axis. Selected helices shown in cartoon form with the remaining part colored in white semi-transparent surface.

**Figure 5:**
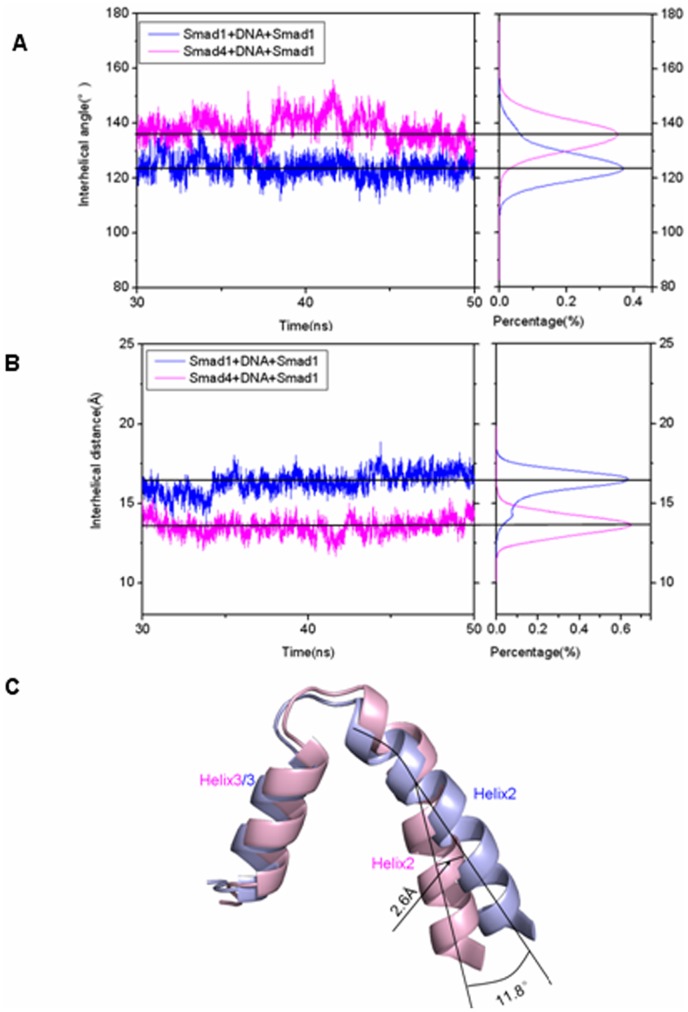
Interhelical angles and distances between helix2 and helix3 in Smad1 for Smad4+DNA+Smad1 and Smad1+DNA+Smad1 models. Interhelical angles (A) and interhelical distances (B) between helix2 and helix3 in Smad1 along with respective integrated distributions for the Smad4+DNA+Smad1 (magenta) and Smad1+DNA+Smad1 (blue) models; (C) The changes of interhelical angles and distances for Smad4+DNA+Smad1 (light pink) and Smad1+DNA+Smad1 (light blue) models.

**Figure 6:**
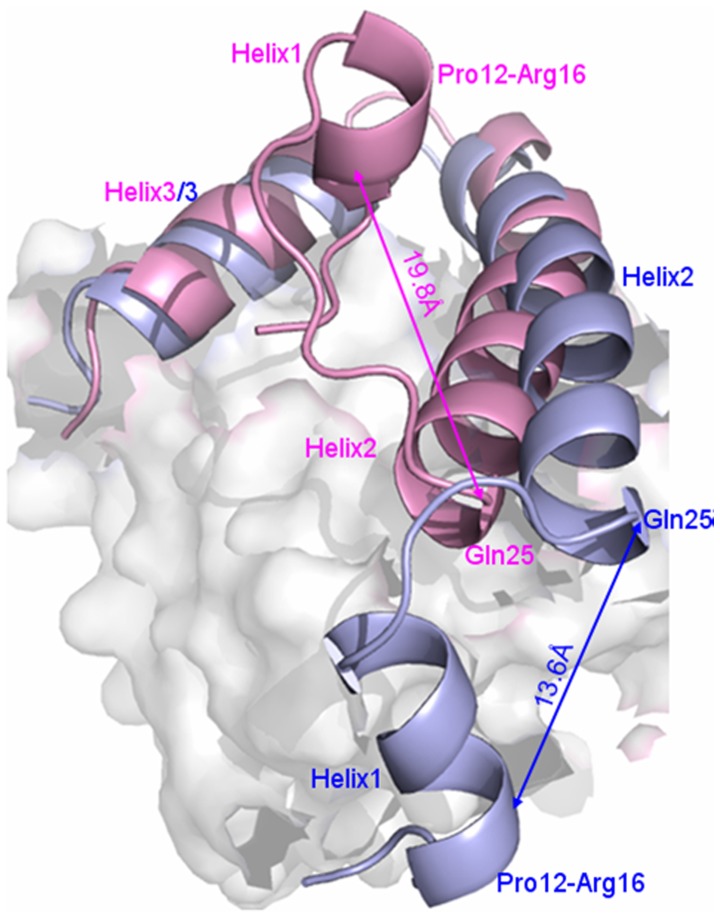
Scheme of conformational changes for helix1 of Smad1 in Smad4+DNA+Smad1 and Smad1+DNA+Smad1 models. Scheme of conformational changes for helix1 of Smad1 in both Smad4+DNA+Smad1 (light pink) and Smad1+DNA+Smad1 (light blue) models with the distances between Pro12-Arg16 residues of helix1 and Gln25 residue of helix2.

**Figure 7:**
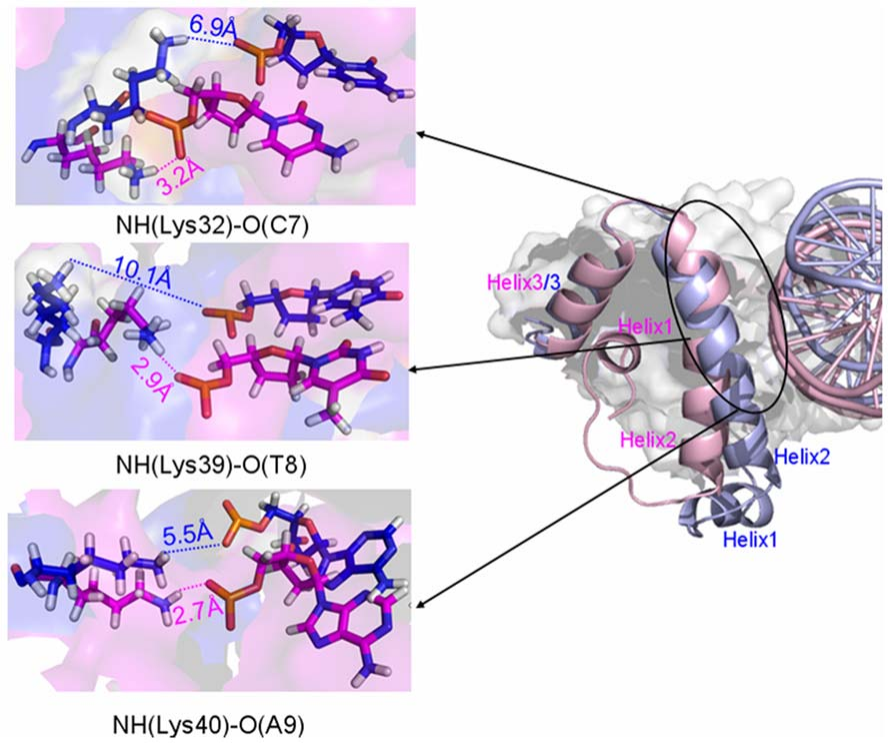
Scheme of hydrogen bonds at Smad1-DNA interfaces for Smad4+DNA+Smad1 and Smad1+DNA+Smad1models. Scheme of hydrogen bonds, NH(Lys32)-O(C7), NH(Lys39)-O(T8) and NH(Lys40)-O(A9), at Smad1-DNA interfaces for Smad4+DNA+Smad1 (light pink) and Smad1+DNA+Smad1 (light blue) models with oxygen atoms of DNA phosphate backbone in red, hydrogen atoms in white, nitrogen atoms in blue and carbon atoms in magenta for Smad4+DNA+Smad1 model, carbon/nitrogen atoms in blue for Smad1+DNA+Smad1 model.

To investigate how the Smad4 protein binding to one side of DNA molecule to modulate Smad1 conformation and thus to increase the affinity of Smad1-DNA interface, visual structural superposition of average structures extracted from the trajectories in the simulations of Smad4+DNA+Smad1 and Smad1+DNA+Smad1 models was shown in Figure 4 and suggested that the interface conformation changes and rearrangement of binding sites in the Smad1-DNA interface caused by Smad4 binding to DNA are notably observed. To investigate the conformational changes at the Smad1-DNA interfaces in the two models, the relative rotational orientations of helix2 and helix3, i.e. interhelical angles and distances, in the Smad1 proteins have been quantitatively examined by using the program INTERHLX. First of all, the interhelical angles and distances of helix2 and helix3 for the Smad1 proteins in the Smad4+DNA+Smad1 and Smad1+DNA+Smad1 models over the simulation times were measured and shown in Figure 5A and B. The calculated data indicate that the interhelical angles and distances of helix2 and helix3 in the Smad1 proteins change from the average values of 55.7° and 16.2 Å in the Smad1+DNA+Smad1 model to those of 43.9° and 13.6 Å in the Smad4+DNA+Smad1 model, respectively (Figure 5C), which presents the conformational change of Smad1-DNA interface due to the Smad4 protein binding to DNA molecule. Especially, the residues Leu17-Lys21 in the helix1 of Smad1 protein in the Smad4+DNA+Smad1 model are regulated to form a loop conformation due to the Smad4 protein binding, and simultaneously the shorted helix1 moves away from helix2 with the increase of average distance between the centroid of residues Leu17-Lys21 in helix1 and the centroid of residue Gln25 in helix2 from 13.6 Å to 19.8 Å compared with the Smad1+DNA+Smad1 model (see Figure 6). As expected, the rearrangement of binding sites and three new-formed hydrogen bonds of NH(Lys32)-O(C7), NH(Lys39)-O(T8) and NH(Lys40)-O(A9) in such changed Smad1-DNA interface for the Smad4+DNA+Smad1 model were observed compared to those for the Smad1+DNA+Smad1 model discussed above (see Figure 7).

#### 1.4 Dynamical fluctuation and correlation analyses revealed the conformation changes of Smad1 protein induced by Smad4 binding

**Figure 8:**
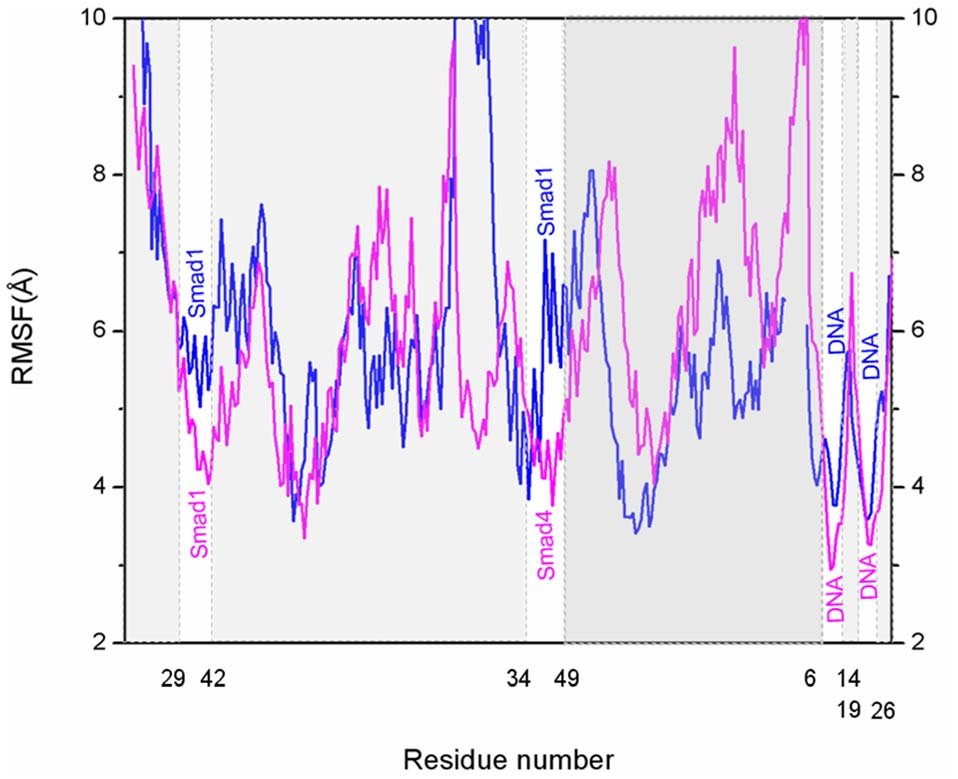
The fluctuations of residues and bases in Smad4+DNA+Smad1 and Smad1+DNA+Smad1 models. The fluctuations of residues and bases in Smad4+DNA+Smad1 (magenta) and Smad1+DNA+Smad1 (blue) models.

To address the interaction of Smad1 protein with DNA molecule and its conformation changes induced by the Smad4 binding via the residue position changes at the Smad1-DNA interface, the dynamics of every residue/base were determined and interpreted by residue/base fluctuations and correlations. The RMSF values were analyzed and shown in Figure 8 for the Smad4/1+DNA+Smad1 model. Most interesting of all, it can be observed that the additional/different hydrogen bonds between the Smad1/Smad4 protein and DNA molecule in the Smad4+DNA+Smad1 model with respect to the Smad1+DNA+Smad1 model can be directly linked to the changes of RMSF values in the fluctuation pattern. Namely, the newly-formed hydrogen bonds between the residues Lys32, Lys39 and Lys40 of Smad1 protein and the bases C7, T8, A9 of DNA, as well as some different hydrogen bonds between the residues Arg38, Ser42 and Lys45 of Smad4 protein and bases T24, A25 and T13 of DNA in the Smad4+DNA+Smad1 model cause the decrease of corresponding fluctuations (see Figure 8), which contributes the stabilization of corresponding contact sites. These results are consistent with the hydrogen bond analysis discussed above. These fluctuation changes between Smad4+DNA+Smad1 and Smad1+DNA+Smad1 models represent the influence of Smad4 binding to DNA on the regulation of Smad1 conformation to favor the affinity of Smad1-DNA interface.

**Figure 9:**
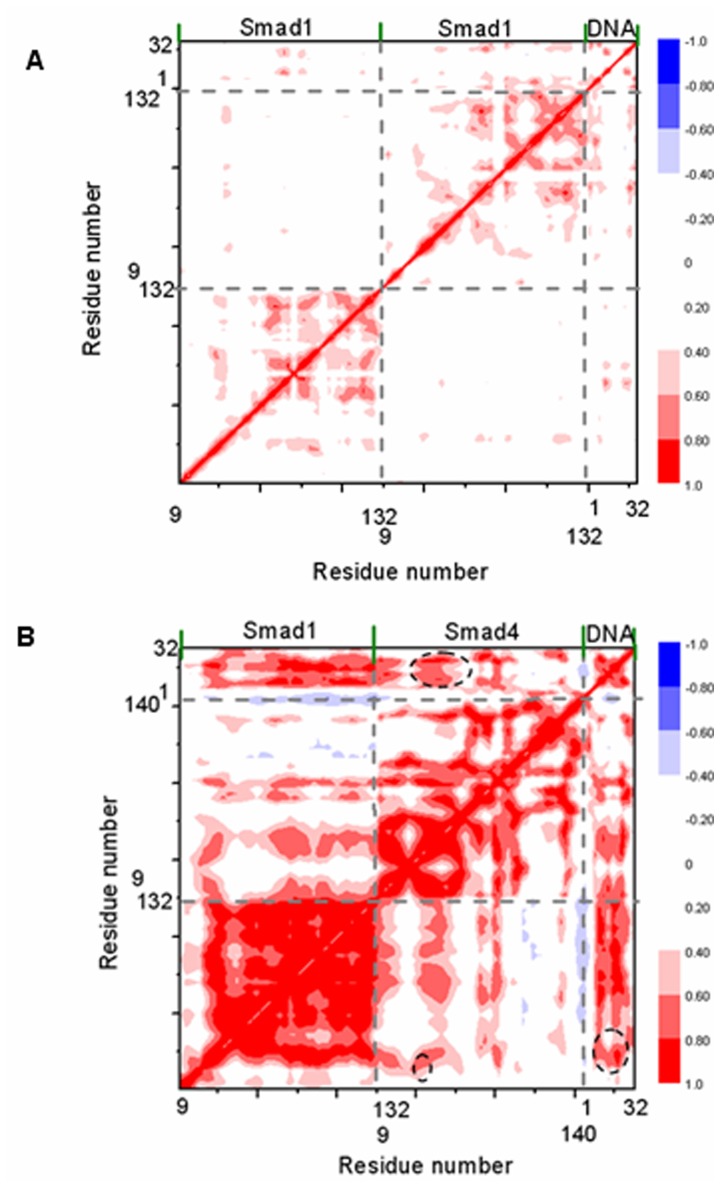
Dynamical cross-correlation maps for Smad1+DNA+Smad1 and Smad4+DNA+Smad1 models. Dynamical cross-correlation maps for Smad1+DNA+Smad1 (A) and Smad4+DNA+Smad1 (B) models with specific sub-regions circled in black.

To further investigate the modulation of Smad4 to the affinity of Smad1-DNA interface, dynamical cross-correlation maps were constructed for the trajectories of Smad4+DNA+Smad1 and Smad1+DNA+Smad1 models and shown in Figure 9. These two maps show the motion correlations between the residues ranging from highly anticorrelated (blue) to highly correlated (red). It can been found from Figure 9B that the large correlated motions of some residues in the helix2 region of Smad4 vs DNA-bound bases at the specific binding sites, at which the formed hydrogen bonds at the Smad4-DNA interface in the Smad4+DNA+Smad1 model differ from those at the Smad1-DNA interface in the Smad1+DNA+Smad1 model, occur remarkably with the large correlated motions of corresponding DNA-bound bases vs Smad1 residues at the additional connect sites, at which the formed additional hydrogen bonds at the Smad1-DNA interface in the Smad4+DNA+Smad1 model differ from those in the Smad1+DNA+Smad1 model. Such results predict the allosteric communication of residue-base between Smad4-DNA and DNA-Smad1 interfaces. Interestingly, the Smad4 binding to DNA amplifies the correlation motions between the Smad4/1 residues and DNA base pairs at the different/additional connection sites in the Smad4/1-DNA interface. Namely, the collective movements of residues Arg38, Ser42, Lys45 at helix2 region of Smad4 correlate the corresponding movements of DNA base pairs T8:A25, A9:T24 and T13:A20, and vice verse in the Smad4+DNA+Smad1 model. Consequently, the collective movements of DNA base pairs C7:G26, T8:A25 and A9:T24 correlate the corresponding movements of residues Lys32, Lys39, Lys40 at helix2 region of Smad1. These results indicate that the binding of Smad4 to DNA might lead to allosteric communication between Smad4-DNA and DNA-Smad1 interfaces via DNA mediator.

Moreover, the residue Arg38 in the helix2 region of Smad4 with the large cross-correlation coefficient also correlates to the residues Leu17-Lys21 at the helix1 region of Smad1 which indicates indirect interactions between Smad4 and Smad1.

### 2. Similar Results Revealed Smad4 Recruiting Smad3 on the Binding Sites of DNA

Similar results can be observed in the case of Smad4/3+DNA+Smad3 model. First of all, Smad3 MH1 domain binding to DNA with the binding free energy of −35.56 kcal·mol^−1^ in the Smad4+DNA+ Smad3 model is energetically favorable over Smad4 MH1 domain with that of −17.34 kcal·mol^−1^ in the Smad4+DNA+Smad4 model, and Smad3 MH1 domain with that of −23.68 kcal·mol^−1^ in the Smad3+DNA+Smad3 model, which is consistent with the experimental result of −10.20 kcal·mol^−1^ analyzed by the dissociation constant K_d_ for the Smad3 MH1+DNA complex [20]. Second, hydrogen bond analysis (see Supplementary Figure S1) indicates that the formations of additional hydrogen bonds between the N-H groups of residues Lys33, Lys40 and Lys41 of helix 2 region in the Smad3 protein and O atoms of DNA phosphate backbone at the bases C7, T8, A9 in the Smad4+DNA+Smad3 model are maintained with the occupancies of 71.33%, 55.91% and 50.25% of simulation times, respectively, compared to the Smad3+DNA+Smad3 model. Interestingly, such two new-formed NH(Lys40)-O(T8) and NH(Lys41)-O(A9) hydrogen bonds in the Smad3-DNA interface induced by the Smad4 binding and located at Lys40 and Lys41 residue sites are consistent with the functional region of the lysine-rich sequence Lys40-Lys41-Leu42-Lys43-Lys44 as the nuclear localization signal in the Smad3 protein reported by the sequence alignment method using CLUSTALX software [24]. Importantly, the measured hydrogen bonds between the residues Arg38, Ser42 and Lys45 of helix2 region in the Smad4 protein and T24, A25 and T13 bases of DNA phosphate backbone maintained with the occupancies of 73.17%, 51.52% and 17.68% of simulation times, respectively, which are consistent with experimental observations of Smad4-specific contact sites with DNA backbone [22]. These results support the recruitment mechanism of Smad4 to Smad3 protein.

## Discussion

### Variations of DNA Groove Parameters Caused by Smad4 Binding

**Figure 10:**
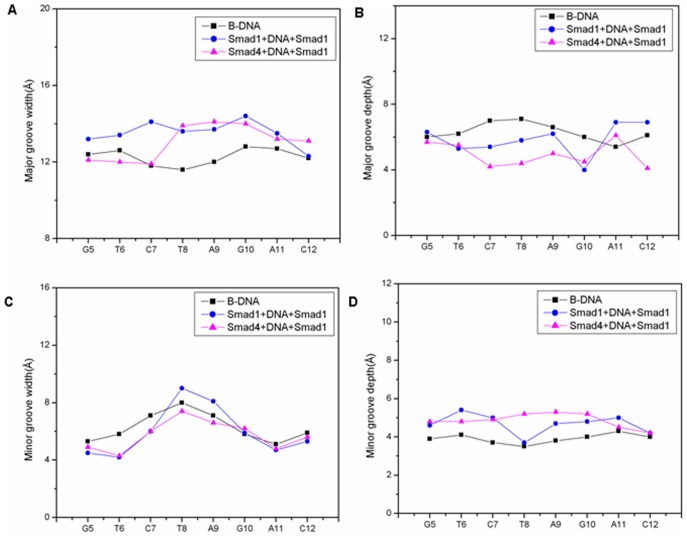
Groove widths and depths of B-DNA, Smad4+DNA+Smad1 and Smad1+DNA+Smad1 models. Groove widths and depths of B-DNA (black line with square), Smad4+DNA+Smad1 (magenta line with up-triangle) and Smad1+DNA+Smad1 (blue line with circle) models. Major groove widths (A), major groove depths (B), minor groove widths (C) and minor groove depths (D) for the time-averaged structures of DNA conformations.

The Smad4 protein binding to DNA backbone is a driving force for the increased interaction between the Smad1 protein and DNA molecule in the Smad4+DNA+Smad1 model, simultaneously inducing DNA conformation distortion. To address the DNA conformation disturbance, the DNA groove parameters along with the DNA palindromic base pairs of G5T6C7T8-A9G10A11C12 for the Smad4+DNA+Smad1, Smad1+DNA+Smad1 and a B-DNA models were analyzed and shown in Figure 10. Interestingly, it is found from Figure 10 that the DNA minor groove width and major groove depth at the critical interaction sites, such as the middle base pairs T8:A25 and A9:T24, decrease with the increases of the minor groove depth and major groove width in the Smad4+DNA+Smad1 model. Namely, the values of DNA major and minor groove widths/depths of 13.6Å/5.8 Å and 8.9 Å/3.7 Å at base pair T8:A25 in the Smad1+DNA+Smad1 model significantly change to the corresponding values of 13.9 Å/4.4 Å and 7.4/5.2 Å, respectively, in the Smad4+DNA+Smad1 model, in which the values of DNA major and minor groove width/depth in the Smad1+DNA+Smad1 model are close to the corresponding values of 11.6 Å/7.1 Å and 8.0 Å/3.5 Å in the B-DNA model. For the base pair A9:T24, the values of DNA major and minor groove widths/depths in the Smad1+DNA+Smad1 model change from the values of 13.7 Å/6.2 Å and 7.9 Å/4.4 Å to 14.1 Å/5.0 Å and 6.7 Å/5.3 Å, respectively, in the Smad4+DNA+Smad1 model, in which, similarly, the values in the Smad1+DNA+Smad1 model are close to the corresponding values of 12.0 Å/6.6 Å and 7.1 Å/3.8 Å in the B-DNA model. Simultaneously, the DNA bend angle with the value of 22.5° toward the DNA minor groove in the Smad1+DNA+Smad1 model is significantly changed to the corresponding value of 38.3° in the Smad4+DNA+Smad1 model, in which the calculated DNA bend angle of 22.5° in the Smad1+DNA+Smad1 model is consistent with the experimental datum of 14.5° [22] and is close to the corresponding value of 9.8° in the B-DNA model. Consequently, it is predicted that the Smad4 protein binding to DNA induced significant DNA conformation distortion at some important binding sites over the Smad1 protein binding.

### Dynamics Analysis of DNA Base Pairs

**Figure 11:**
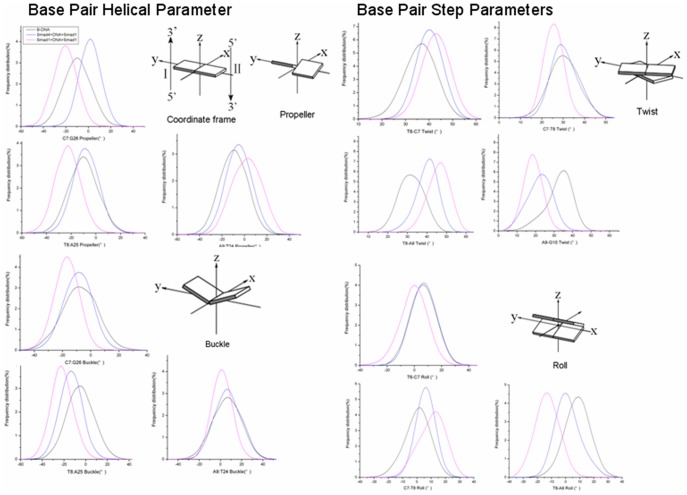
Frequency distributions of DNA duplex helical parameters for B-DNA, Smad1+DNA+Smad1 and Smad4+DNA+Smad1 models. Selected frequency distributions of representative DNA duplex helical parameters for B-DNA (black line), Smad1+DNA+Smad1 (blue line), and Smad4+DNA+Smad1 (magenta line) models.

To further investigate the relationship between DNA groove-parameters changes and DNA base pair motions due to DNA bound by Smad4 protein, the frequency distributions of DNA base-pair dynamics for the Smad4+DNA+Smad1 and Smad1+DNA+Smad1 models were analyzed and shown in Figure 11 along with the distribution patterns of the undamaged B-DNA model. There were the distinct differences of several motions, such as propeller, buckle, roll and twist, identified from DNA base-pair helical dynamics at the critical interaction sites C7:G26, T8:A25 and A9:T24. For example, the propeller and buckle motions with the values of ∼−26° and ∼−24° at the base-pair T8:A25 in the Smad4+DNA+Smad1 model is changed away by ∼−8° and ∼−15° from those in the Smad1+DNA+Smad1 model, and by ∼−9° and ∼−2° in the B-DNA model. The high degree of propeller twist at the base pairs C7:G26, T8:A25 and A9:T24 in the Smad4+DNA+Smad1 model significantly amplifies the effect of base-stacking between neighbouring bases, which contributes to the narrowing of minor groove. Simultaneously, these changed values of propeller and buckle motions represent the changes of orientations of base pairs C7:G26, T8:A25 and A9:T24 in the Smad4+DNA+Smad1 model, which causes the O atoms of DNA phosphate backbone at the bases C7, T8 and A9 close to N-H groups of residues Lys32, Lys39, Lys40 of Smad1 protein to form new hydrogen bonds at the Smad1-DNA interface. Moreover, the roll motion with the values of ∼0°, ∼15°, ∼−15°, respectively, at the base-pair steps T6–C7, C7–T8 and T8–A9 in the Smad4+DNA+Smad1 model are changed away from those of ∼10°, ∼5° and ∼0° in the Smad1+DNA+Smad1 model, and from those of ∼6°, ∼0° and ∼10 ° in the B-DNA model; based on the anticorrelation of roll and twist motions, the twist angle at the center of such sequence G5T6C7T8-A9G10A11C12 increases from the value of ∼40° in the Smad1+DNA+Smad1 to that of ∼48° in the Smad4+DNA+Smad1 model compared with that of 33.7° in the B-DNA model, which contributes to the larger bend of DNA helix toward the minor groove for the Smad4+DNA+Smad1 model over the Smad1+DNA+Smad1 model. Summarily, the DNA base-pair motions result in the DNA groove parameter changes and hydrogen bond formations at the Smad1-DNA interface in the Smad4+DNA+Smad1 model.

### Allosteric Communication from the Smad4-DNA Interface to DNA-Smad1 Interface

To understand fully the allosteric communication from Smad4-DNA interface to DNA-Smad1 interface in the Smad4+DNA+Smad1 model via DNA molecule, we explore relationship between the residue/base motion correlations and conformational changes at such two interfaces. As expected, it can be seen from motion correlation analysis that the Smad4 residues Arg38, Ser42, Lys45 correlate to DNA base pairs T8:A25, A9:T24 and T13:A20 with large cross-correlation coefficients at the Smad4-DNA interface; simultaneously, at the DNA-Smad1 interface the DNA base pairs C7:G26, T8:A25 and A9:T24 correlate to the residues Lys32, Lys39, Lys40 at the helix2 region of Smad1. Such correlation analysis presents that the allosteric communication between the two Smad4-DNA and DNA-Smad1 interfaces will occur at these key sites. According to the visual analysis of average structures in our MD simulations, it can be seen that the Smad4-specific residues Arg38 and Ser42 of helix2 interact with the base pairs G10:C23, A9:T24 and T8:A25 at the Smad4-DNA interface with the hydrogen bond formations between the N-H groups of residues Arg38, Ser42 at the helix2 of Smad4 protein and O atoms of DNA phosphate backbone at the bases C23, T24, A25 with the occupancies of 37.32%, 12.41% and 62.71% of simulation times, simultaneously inducing the DNA base pair motions of propeller, buckle, roll and twist; such DNA base pair motions cause consequentially some additional hydrogen bond formations at the Smad1-DNA interface in the Smad4+DNA+Smad1 model, such as the hydrogen bonds between the N-H groups of residues Lys32, Lys39, Lys40 of Smad1 and O atoms of DNA phosphate backbone at the base pairs C7:G24, T8:A25, A9:T24 with the occupancies of 58.6%, 31.78% and 35.74% of simulation times. Moreover, the conformation changes at the Smad1-DNA interface with the residues Leu17-Lys21 in the helix1 of Smad1 regulating to a loop region and the decrease of interhelical angle/distance between the helix2 and helix3 in the Smad1 protein also present the effect of allosteric communication between the two interfaces. Similar results for the Smad4+DNA+Smad3 model can be found and shown in Supplementary Materials (see Supplementary Figure S2, S3, S4, S5, S6). Summarily, our modeling studies suggest that the Smad4 protein binding to DNA induces the distortion of DNA, and consequently, the increase of interaction of the Smad1/Smad3 protein with DNA molecule via the allosteric communication between the Smad4-DNA interface and DNA-Smad1/Smad3 interface, which theoretically explains the mechanism of cooperative recruitment of Smad4 protein to Smad1/Smad3 in the nucleus. Moreover, based on the correlation of the residue Arg38 in Smad4 with the residues Leu17-Lys21 in Smad1, the conformation difference of Arg38 in Smad4 from Lys32 at the corresponding site in Smad1 might contribute to indirectly regulating the residues Leu17-Lys21 in the helix1 of Smad1 to a loop region.

### Conclusions

Molecular dynamics simulations, free energy calculations and DNA dynamics analysis for a series of constructed Smadn+DNA+Smadn (n = 1, 3, 4) models have been performed to address the interaction characteristics of Smadn (n = 1, 3, 4) with DNA molecule, further to understand the mechanism of cooperative recruitment of Smad4 protein to Smad1 or Smad3 on DNA. The results demonstrated that the Smad4 protein binding to DNA molecule is energetically favorable to increase the affinity of Smad1/3 with DNA molecule in the Smad4+DNA+Smad1/3 model. The visual structure analysis indicates the formations of some additional hydrogen bonds at the Smad1/3-DNA interface, which supports the energetic observation. The Smad4 binding heteromerously to DNA molecule in the Smad4+DNA+Smad1/3 model also induce the conformation regulation at the Smad1/3-DNA interface, with the decrease of interhelical angle/distance between the helix2 and helix3 in the Smad1/3 protein. It has been found from the residue/base motion correlation analysis that the residues Arg38, Ser42 and Lys45 of Smad4 with large cross-correlation coefficients correlate to the DNA base pairs T8:A25, A9:T24 and T13:A20 at the Smad4-DNA interface; simultaneously, such base pairs correlate further to the residues Lys32, Lys39 and Lys40 at the Smad1-DNA interface, or to the residues Lys33, Lys40 and Lys41 at the Smad3-DNA interface. As expected, based on such correlation and DNA dynamics analyses, DNA base pair motions of propeller, buckle, roll and twist at such correlated interaction sites C7:G26, T8:A25 and A9:T24 allosterically communicate the interactions to the Smad1/3-DNA interface with the formations of additional hydrogen bonds between the O atoms of DNA phosphate backbone at the bases C7, T8 and A9 and N-H groups of residue Lys32, Lys39 and Lys40, or the residues Lys33, Lys40 and Lys41 in the Smad1/3 protein. Our simulation results provide useful insights into understanding the Smad4 protein binding to DNA molecule, as a binding vehicle, to modulate DNA molecule conformation, consequently increasing the interaction of DNA with the Smad1/3 protein, which theoretically explains the mechanism of cooperative recruitment of the Smad4 protein to the Smad1/3 protein via DNA-mediated indirect readout mode.

## Supporting Information

Figure S1 **The hydrogen bonds at Smad3-DNA and Smad4-DNA interfaces.** The occupancies of hydrogen bonds at Smad3-DNA interfaces in Smad4+DNA+Smad3 (magenta) and Smad3+DNA+Smad3 (blue) models on the left, respectively; and the occupancies of hydrogen bonds at Smad4-DNA interface in the Smad4+DNA+Smad3 model (magenta) and at Smad3-DNA interface in the Smad3+DNA+Smad3 model (blue) on the right, respectively.
(TIF)

Figure S2 **The superpositions of average structures for Smad4+DNA+Smad3 and Smad3+DNA+Smad3 models.** The visual superpositions for average structures of Smad4+DNA+Smad3 (light blue) and Smad3+DNA+Smad3 (light pink) models as viewed parallel to DNA axis. Selected helices shown in canton form with the remaining part colored in white semi-transparent surface.
(TIF)

Figure S3 **Interhelical angles and distances between helix2 and helix3 in Smad3 for Smad4+DNA+Smad3 and Smad3+DNA+Smad3 models.** Interhelical angles (A) and interhelical distances (B) between helix2 and helix3 of Smad3 along with respective integrated distributions for the Smad4+DNA+Smad3 (magenta) and Smad3+DNA+Smad3 (blue) models; (C) The changes of interhelical angles and distances for Smad4+DNA+Smad3 (light pink) and Smad3+DNA+Smad3 (light blue) models.
(TIF)

Figure S4 **Dynamical cross-correlation maps for Smad3+DNA+Smad3 and Smad4+DNA+Smad3 models.** Dynamical cross-correlation maps for Smad3+DNA+Smad3 (A) and Smad4+DNA+Smad3 (B) models with specific sub-regions circled in black.
(TIF)

Figure S5 **Groove widths and depths of B-DNA, Smad4+DNA+Smad3 and Smad3+DNA+Smad3 models.** Groove widths and depths of B-DNA (black line with square), Smad4+DNA+Smad3 (magenta line with up-triangle) and Smad3+DNA+Smad3(blue line with circle) models. Major groove widths (A), major groove depths (B), minor groove widths (C) and minor groove depths (D) for the time-averaged structures of DNA conformations.
(TIF)

Figure S6 **Frequency distributions of DNA duplex helical parameters for B-DNA, Smad3+DNA+Smad3 and Smad4+DNA+Smad3 models.** Selected frequency distributions of representative DNA duplex helical parameters for B-DNA (black line), Smad3+DNA+Smad3 (blue line), and Smad4+DNA+Smad3 (magenta line) models.
(TIF)

Text S1 **Molecular dynamics simulation protocols used in this work.**
(DOC)

Text S2 **MM-PBSA calculation for free energy.**
(DOC)

Text S3 **DNA helical parameter analysis of trajectories.**
(DOC)
