# Supplementary figures and images for: Cooperative Assembly of Co-Smad4 MH1 with R-Smad1/3 MH1 on DNA: A Molecular Dynamics Simulation Study
Source: PLoS One. 2013 Jan 10;8(1):e53841. doi: 10.1371/journal.pone.0053841 (PMC3542330; doi:10.1371/journal.pone.0053841)

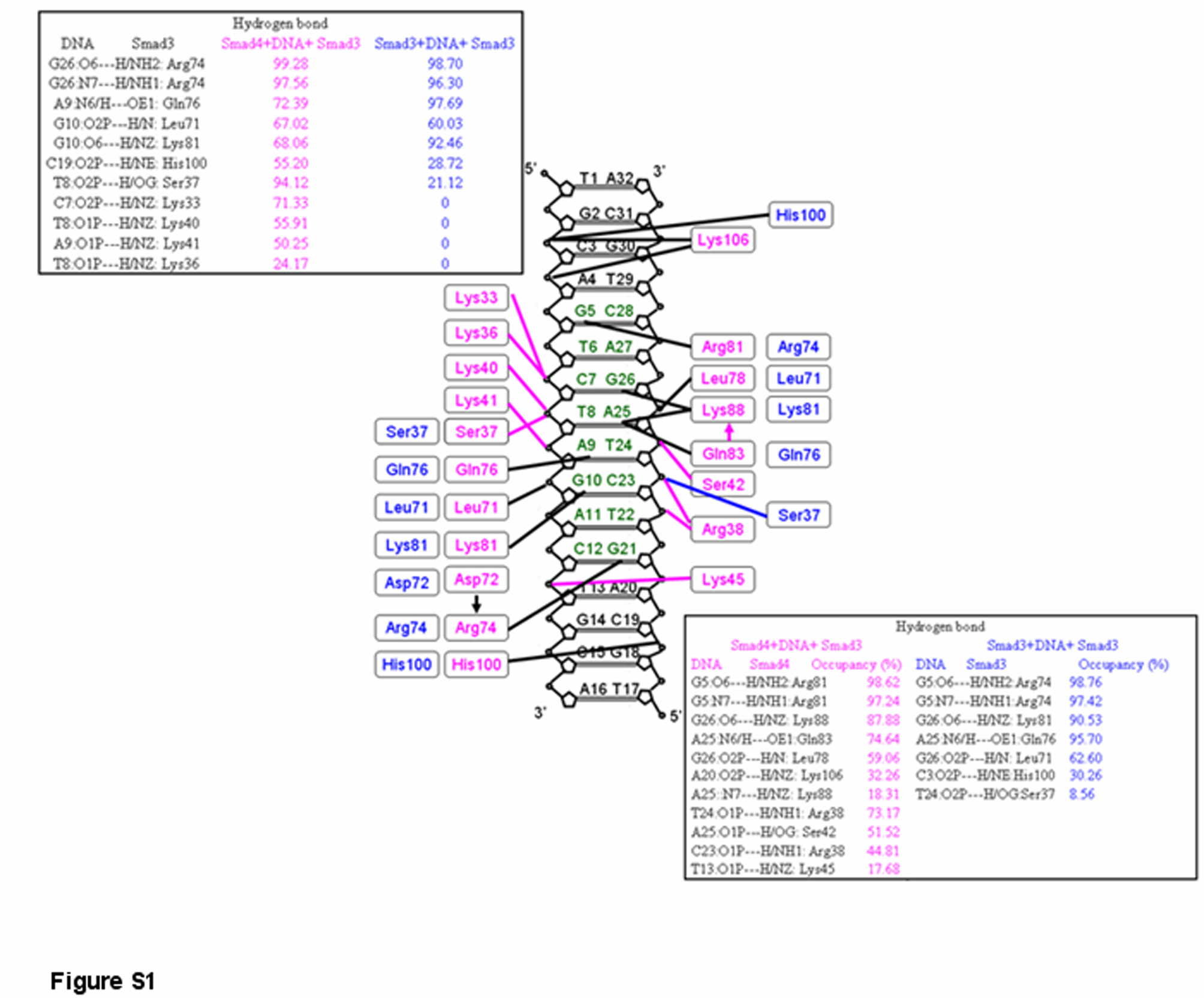

Supplement: Figure S1 — The hydrogen bonds at Smad3-DNA and Smad4-DNA interfaces. The occupancies of hydrogen bonds at Smad3-DNA interfaces in Smad4+DNA+Smad3 (magenta) and Smad3+DNA+Smad3 (blue) models on the left, respectively; and the occupancies of hydrogen bonds at Smad4-DNA interface in the Smad4+DNA+Smad3 model (magenta) and at Smad3-DNA interface in the Smad3+DNA+Smad3 model (blue) on the right, respectively. (TIF) [file pone.0053841.s001.tif]

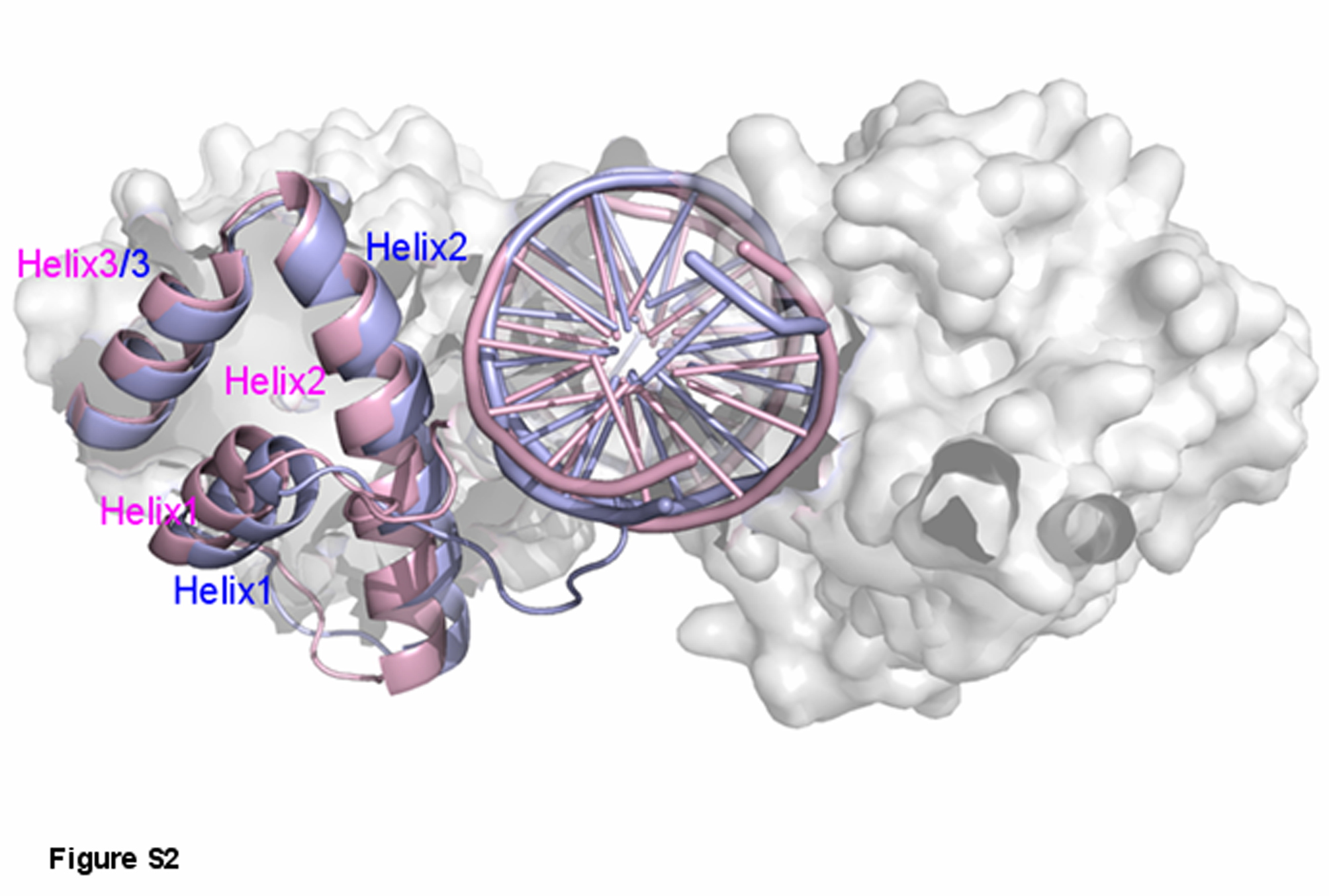

Supplement: Figure S2 — The superpositions of average structures for Smad4+DNA+Smad3 and Smad3+DNA+Smad3 models. The visual superpositions for average structures of Smad4+DNA+Smad3 (light blue) and Smad3+DNA+Smad3 (light pink) models as viewed parallel to DNA axis. Selected helices shown in canton form with the remaining part colored in white semi-transparent surface. (TIF) [file pone.0053841.s002.tif]

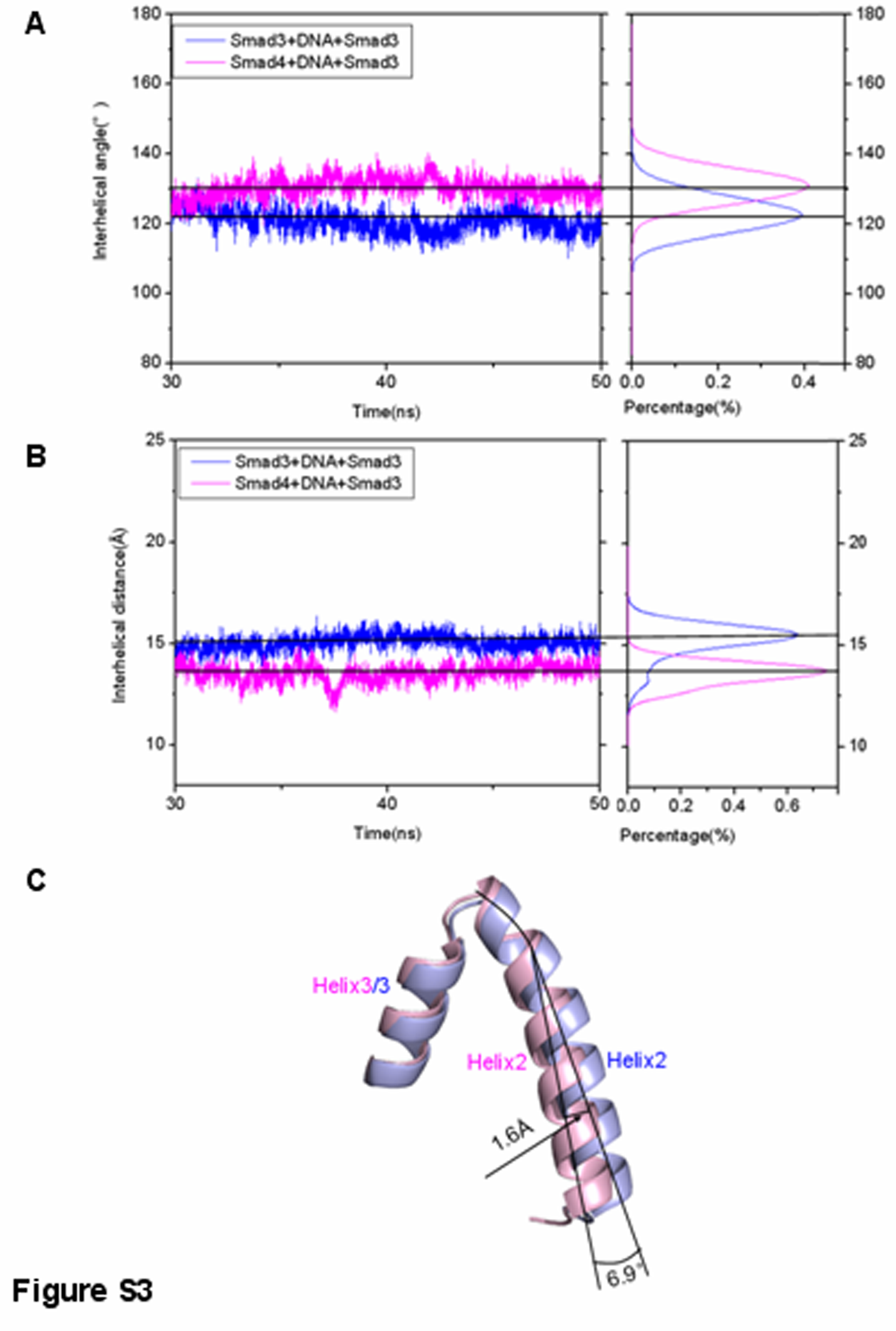

Supplement: Figure S3 — Interhelical angles and distances between helix2 and helix3 in Smad3 for Smad4+DNA+Smad3 and Smad3+DNA+Smad3 models. Interhelical angles (A) and interhelical distances (B) between helix2 and helix3 of Smad3 along with respective integrated distributions for the Smad4+DNA+Smad3 (magenta) and Smad3+DNA+Smad3 (blue) models; (C) The changes of interhelical angles and distances for Smad4+DNA+Smad3 (light pink) and Smad3+DNA+Smad3 (light blue) models. (TIF) [file pone.0053841.s003.tif]

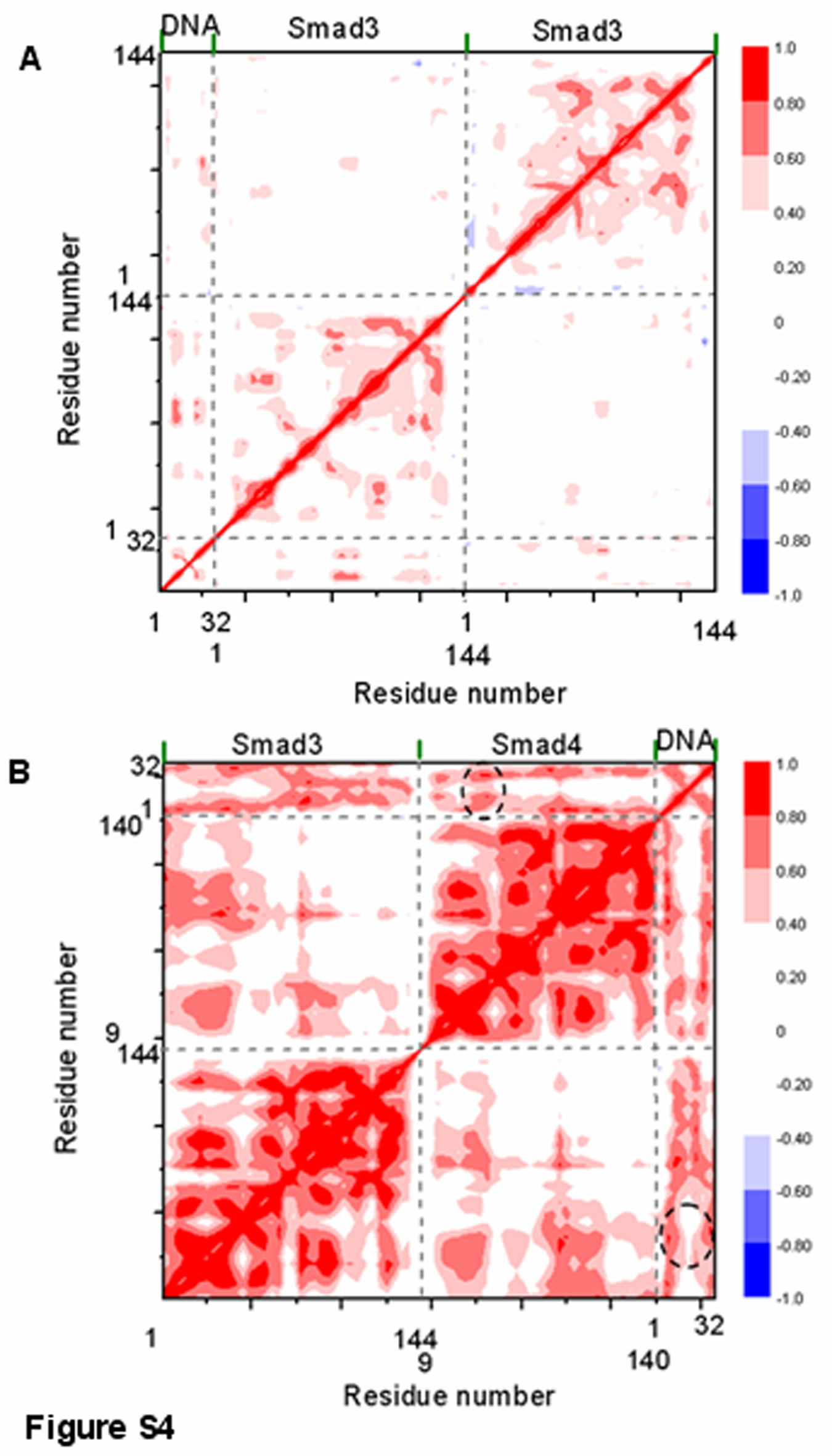

Supplement: Figure S4 — Dynamical cross-correlation maps for Smad3+DNA+Smad3 and Smad4+DNA+Smad3 models. Dynamical cross-correlation maps for Smad3+DNA+Smad3 (A) and Smad4+DNA+Smad3 (B) models with specific sub-regions circled in black. (TIF) [file pone.0053841.s004.tif]

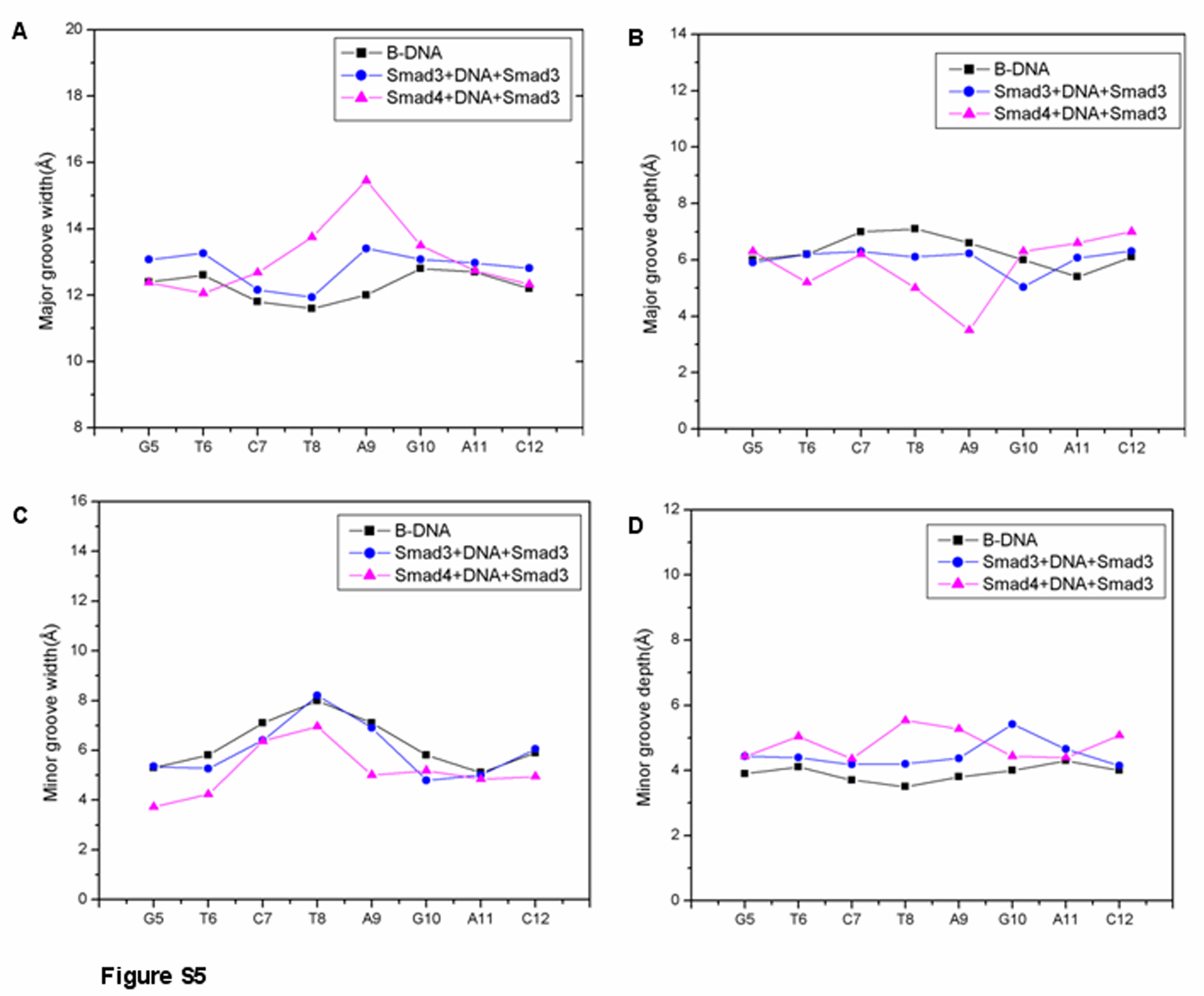

Supplement: Figure S5 — Groove widths and depths of B-DNA, Smad4+DNA+Smad3 and Smad3+DNA+Smad3 models. Groove widths and depths of B-DNA (black line with square), Smad4+DNA+Smad3 (magenta line with up-triangle) and Smad3+DNA+Smad3(blue line with circle) models. Major groove widths (A), major groove depths (B), minor groove widths (C) and minor groove depths (D) for the time-averaged structures of DNA conformations. (TIF) [file pone.0053841.s005.tif]

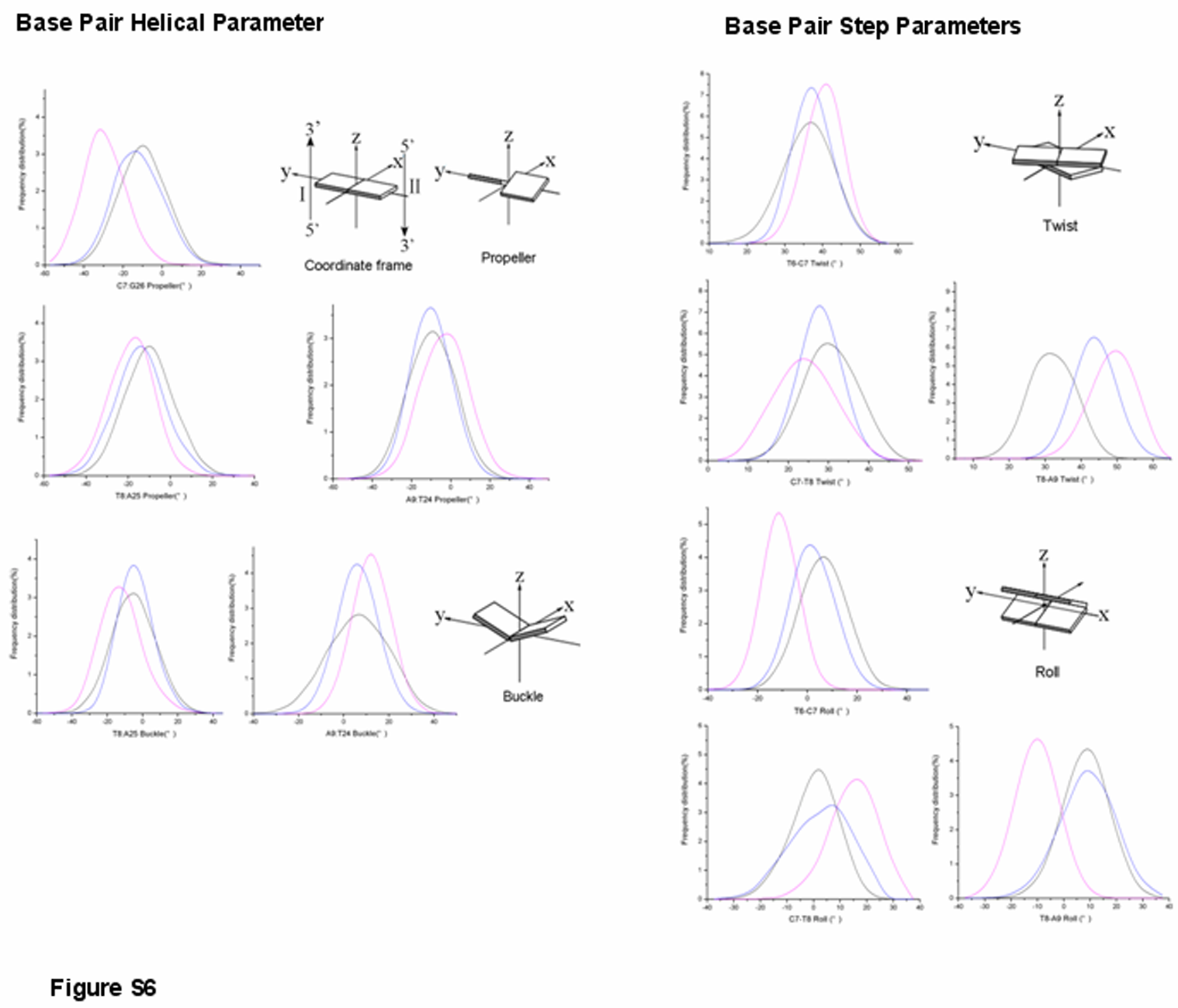

Supplement: Figure S6 — Frequency distributions of DNA duplex helical parameters for B-DNA, Smad3+DNA+Smad3 and Smad4+DNA+Smad3 models. Selected frequency distributions of representative DNA duplex helical parameters for B-DNA (black line), Smad3+DNA+Smad3 (blue line), and Smad4+DNA+Smad3 (magenta line) models. (TIF) [file pone.0053841.s006.tif]
