# Supplementary material for: Cooperative Assembly of Co-Smad4 MH1 with R-Smad1/3 MH1 on DNA: A Molecular Dynamics Simulation Study
Source: PLoS One. 2013 Jan 10;8(1):e53841. doi: 10.1371/journal.pone.0053841 (PMC3542330; doi:10.1371/journal.pone.0053841)
Supplement: Text S1 — Molecular dynamics simulation protocols used in this work. (DOC) [file pone.0053841.s007.doc]

**Text S1**

**Molecular dynamics simulation protocols**

All MD simulations were carried out using the AMBER9 package [1] with a classical AMBER parm99 [2,3] together with the parmbsc0 refinement [4] and gaff [5] force field parameters. The protocol for all MD simulations is described herein as follows: (1) the systems were energetically minimized to remove unfavorable contacts. Four cycles of minimizations were performed with 5000 steps of each minimization and harmonic restraints on DNA and the Smad1/3/4 MH1 protein from 100 kcal·mol^-1^·Å^-2^, 75 kcal·mol^-1^·Å^-2^, 50 kcal·mol^-1^·Å^-2^ to 25 kcal·mol^-1^·Å^-2^, which means that the restraints were relaxed stepwisely by 25 kcal·mol^-1^·Å^-2^ per cycle. The fifth cycle consists of 10000 steps of unrestrained minimization before heating process. The cutoff distance used for the non-bonded interactions was 10 Å. The SHAKE algorithm [6] was used to restrain the bonds containing hydrogen atoms. (2) Each energy-minimized structure was heated over 120 ps from 0 to 300 K (with a temperature coupling of 0.2 ps), while the positions of DNA and the Smad1/3/4 MH1 protein were restrained with a small value of 25 kcal·mol^-1^·Å ^-2^. The constant volume was maintained during the processes. (3) The unrestrained equilibration of 200 ps with constant pressure and temperature conditions was carried out for each system. The temperature and pressure were allowed to fluctuate around 300 K and 1 bar, respectively, with the corresponding coupling of 0.2 ps. For each simulation, an integration step of 2 fs was used. (4) Finally, production runs of 50 ns were carried out by following the same protocol. A time point after thermal equilibration of 200 ps in each simulation was selected as a starting point for data collection. During the production runs, 15000-25000 structures for a simulation were saved for post-processing by uniformly sampling the trajectory.

**References**

1. Case DA, Darden TA, Cheatham TE III, Simmerling CL, Wang JM, et al. (2006) University of California, San Francisco.

2. Duan Y, Wu C, Chowdhury S, Lee MC, Xiong G, et al. (2003) A point-charge force field for molecular mechanics simulations of proteins based on condensed-phase quantum mechanical calculations. . J Comput Chem 24: 1999-2012.

3. Lee MC, Duan Y (2004) Distinguish protein decoys by Using a scoring function based on a new AMBER force field, short molecular dynamics simulations, and the generalized born solvent model. Proteins 55: 620-634.

4. Perez A, Marchan I, Svozil D, Sponer J, Cheatham TE III, et al. (2007) Refinement of the AMBER force field for nucleic acids: improving the description of alpha/gamma conformers. Biophys J 92: 3817-3829.

5. Wang J, Wolf RM, Caldwell JW, Kollamn PA, Case DA (2004) Development and testing of a general Amber force field. J Comput Chem 25: 1157-1174.

6. Miyamoto S, Kollman PA (1992) Settle: An analytical version of the SHAKE and RATTLE algorithm for rigid water models. J Comput Chem 13: 952-962.
