# Supplementary material for: Cooperative Assembly of Co-Smad4 MH1 with R-Smad1/3 MH1 on DNA: A Molecular Dynamics Simulation Study
Source: PLoS One. 2013 Jan 10;8(1):e53841. doi: 10.1371/journal.pone.0053841 (PMC3542330; doi:10.1371/journal.pone.0053841)
Supplement: Text S2 — MM-PBSA calculation for free energy. (DOC) [file pone.0053841.s008.doc]

**Text S2**

**MM-PBSA calculation for free energy**

Energetic post-process of single-trajectory/triplet-trajectory was performed for each MM-PBSA calculation by using the MM-PBSA module of AMBER9 program through molecular mechanics and a continuum solvent model [1]. In MM-PBSA calculation, *G*_np/solv_ is non-polar solvation free energy, which was calculated by using a solvent accessible surface area (SASA) as follows:

*G*_np/solv_ = rSASA + b

The SASA is the solvent-accessible surface area, and is estimated using Sanner’s algorithm implemented in the Molsurf program in AMBER9 [2] with a probe radius of 1.4 Å. The surface tension proportionality constant (r) and the free energy of non-polar solvation for a point solute (b) are set to 0.00542 kcal mol^-1^ Å^-2^ and 0.92 kcal mol^-1^, respectively.

For each of the three homomeric Smad4+DNA+Smad4, Smad1+DNA+Smad1, Smad3+DNA+Smad3 and other two heteromeric Smad4+DNA+Smad1 and Smad4 +DNA+Smad3 models, the last 20 ns trajectory of the production dynamics stage was used for binding free energy calculations of MM-PBSA, namely, the 2000 snapshots of each model at a 10-ps interval for computation of enthalpy and 20 snapshots at 1000-ps intervals for computation of entropy. The following equation has been employed to calculate the standard error (SE) of the binding free energy shifts:

$$SE=\frac{RMSF}{\sqrt{N}}$$

Where *N* is the number of snapshots chosen in the calculations; *RMSF* is the root-mean-square fluctuation of the calculated Δ*G*_binding_ values associated with all snapshots [3]

**References**

1. Case DA, Darden TA, Cheatham ITE, Simmerling CL, Wang JM, et al (2006) University of California, San Francisco.

2. ML C (1983) Analytical molecular surface calculation. J Appl Cryst 16: 548-558.

3. Hao G-F, Yang G-F, and Zhan C-G (2010) Computational Mutation Scanning and Drug Resistance Mechanisms of HIV-1 Protease Inhibitors. J Phys Chem B 114:9663–9676.
